# Production of commercially important enzymes from *Bacillus licheniformis* KIBGE-IB3 using date fruit wastes as substrate

**DOI:** 10.1186/s43141-020-00060-8

**Published:** 2020-08-31

**Authors:** Fatima Aslam, Asma Ansari, Afsheen Aman, Granaz Baloch, Gissed Nisar, Abdul Hameed Baloch, Haneef Ur Rehman

**Affiliations:** 1 Department of Natural and Basic Sciences, University of Turbat, Turbat, 92600 Pakistan; 2 Dr. A. Q. Khan Institute of Biotechnology and Genetic Engineering (KIBGE), University of Karachi, Karachi, Pakistan; 3 Faculty of Agriculture, Lasbela University of Agriculture, Water and Marine Sciences (LAWMS), Uthal, Pakistan

**Keywords:** *Bacillus licheniformis*, Date fruit wastes, Enzymes, Solid state fermentation

## Abstract

**Background:**

Pakistan is one of the top five date fruit-producing countries and produced more than 30% wastes in picking, packing, storage, and commercialization stages. The date fruit wastes are usually considered inedible for humans and only used for livestock feed. In current research, *Bacillus licheniformis* KIBGE-IB3 was screened for pectinase, xylanase, cellulase, and amylase production using date fruit wastes as substrate through solid state fermentation.

**Results:**

The *B. licheniformis* KIBGE-IB3 produced higher concentration of pectinase using date fruit wastes as substrate as compared to amylase, cellulase, and xylanase. *B. licheniformis* KIBGE-IB3 produced maximum pectinase using 5.0 g/dl date fruit wastes and 0.5 g/dl yeast extract. *B. licheniformis* KIBGE-IB3 required pH 7.0, 37 °C incubation temperature, and 72 h incubation period for maximum production of pectinase.

**Conclusion:**

It has been concluded that date fruit waste is a good source of biomass and can be utilized for the commercial production of pectinase.

## **Background**

Enzymes due to its catalytic properties have been used for various industrial processes including food, diagnosis, pharmaceutical, and textile. Currently more than 4000 enzymes are identified and 200 are used for industrial preparations. Majority of enzymes are carbohydrate-based due to its natural availability for living organisms as biomass. Microbial enzymes are usually preferred for commercial applications due to technical easiness to obtain, economical feasibility, and easy product recovery and optimization. The cost of microbial enzymes depend on the substrates used to induce the enzyme production during fermentation [1].

*B. subtilis*, *B. stearothermophillus*, *B. licheniformis*, and *B. amyloliquefaciens* are reported extensively as industrial enzyme producer of enzymes due to generally recognized as safe [2–4]. Solid state fermentation technology (SSF) using biological wastes as substrate for production enzymes by microbial strains much more cost effective technique as compared to submerged fermentation (SmF) technology [4, 5].

Date palms are cultivated in many region countries of the Middle East and the Central Asia, and its fruits have been used as stable food. Approximately more than 120 million date palm trees are cultivated on 800,000 ha in more than 30 countries globally [6, 7]. Pakistan is one of the top five date fruit-producing countries. More than 30% wastes were generated in picking, packing, storage, and commercialization stages of date fruit preparation. It has been reported that the date palm trees produced 4.5 million tons wastes across the world [8]. These wastes mostly are left in agricultural lands and burned [9].

In the current research, date fruit wastes were screened as substrate for the production of commercially important enzymes such as pectinase, amylase, cellulase, and xylanase through solid state fermentation (SSF). Furthermore, the production parameters of SSF were optimized for maximum biosynthesis of pectinase using date fruit wastes.

## Methods

### Collection of date fruit wastes

Date palm fruit wastes were collected from the local farms. The collected date palm fruit waste was kept in a large tray and cleaned, and the dust and dirt particles were removed. Date palm fruit waste was air-dried, covered with a muslin cloth, and kept at room temperature for 48 h.

### Bacterial strains

The bacterial strain *Bacillus licheniformis* KIBGE-IB3 was obtained from the culture bank of Industrial Biotechnology Section, Dr. A. Q. Khan Institute of Biotechnology and Genetic Engineering (KIBGE) University of Karachi, which was previously isolated from indigenous source [10].

### Solid state fermentation

Solid state fermentation was done by fermentation of *B. lichemiformis* KIBGE-IB3 using a 250-ml Erlenmeyer flask containing 5% date fruit wastes as substrate in a 250-ml flask (sterilized at 120 °C/40 min) and inoculated with 10 ml aliqouts of *B. lichemiformis* KIBGE-IB3 in medium containing peptone 0.025%, yeast extract 0.015%, MgSO_4_ 0.001%, and K_2_HPO_4_ 0.0005% at pH 7.0. The fermentation was carried out at 37 °C for 72 h, and after that, the solid fermented material was mixed with 30 ml distilled water and stirred for 30 min. Then, the mixture was centrifuged 4000 rpm for 20 min and the supernatant was used for crude enzyme.

### Screening of date fruit wastes for pectinase, xylanase, cellulase, and amylase production

*Bacillus licheniformis* KIBGE-IB3 was screened for pectinase, xylanase, cellulase, and amylase production using date fruit wastes as substrate. Four different media with different compositions (Table 1) were used specifically for amylase, pectinase, xylanase, and cellulase production. Equal size of inoculums was used in each media for each specific enzyme production and fermented at pH 7.0 and 37 °C for 72 h.

**Table 1:** Media composition for various enzyme production

Chemicals	Medium-1 (pectinase) (g/100ml)	Medium-2 (cellulase) (g/100ml)	Medium-3 (xylanase)	Medium-4 (amylase)
Date fruit wastes	5 g	5 g	5 g	5 g
Peptone	0.025 g	0.025 g	0.025 g	0.025 g
Yeast extract	0.015 g	0.015 g	0.015 g	0.015 g
MgSO_4_	0.001 g	0.001 g	0.001 g	0.001 g
K_2_HPO_4_	0.0005 g	0.0005 g	0.0005 g	0.0005 g
	Pectin 0.05 g	CMC 0.05 g	Xylan 0.05 g	Starch 0.05 g

### Analysis of physical and chemical parameters of SSF for pectinase biosynthesis

The chemical and physical parameters of SSF including medium composition, temperature, pH, and incubation period were optimized for pectinase production. The concentration effect of date fruit wastes, nitrogen source, fermentation period, temperature, and pH were analyzed by fermenting the *B. licheniformis* KIBGE-IB3 through one variable at time approach.

### Enzyme assays

The enzyme assays of pectinase, xylanase, cellulase, and amylase were performed by incubation of crude enzyme with 1% substrate specific for each enzyme (pectin, xylan, cellulose, and starch) for a defined time period, and after that, the concentration of the end product (galacturonic acid, xylose, and glucose) produced was measured by DNS method. The unit definition of enzymes is “quantity of enzyme needed to produce one μmol of product per minutes under defined physical and chemical conditions”.

## Results

### Using of date fruit wastes for the production of enzymes

*Bacillus licheniformis* KIBGE-IB3 were inoculated in four different media specific for each enzyme production with the addition of 5% date fruit wastes in each medium. It was observed that *Bacillus licheniformis* KIBGE-IB3 produced pectinase, xylanase, amylase, and cellulase using date fruit wastes but pectinase production was higher as compared to the others enzymes (Table 2).

**Table 2:** Screening of date fruit wastes for the production of pectinase, xylanase, cellulase, and amylase by *Bacillus licheniformis* KIBGE-IB3

S. No.	Enzymes	Enzyme activity (U/ml/min)
**1**	Amylase	23,383
**2**	Xylanase	1341
**3**	Cellulase	821
**4**	Pectinase	30,600

### Analysis of physical and chemical parameters of solid state fermentation

The fermentation parameters of SSF technology was optimized for pectinase production by *B. licheniformis* KIBGE-IB3 using one variable at time approach.

#### Influence of date fruit waste concentration on pectinase production

**Fig. 1:**
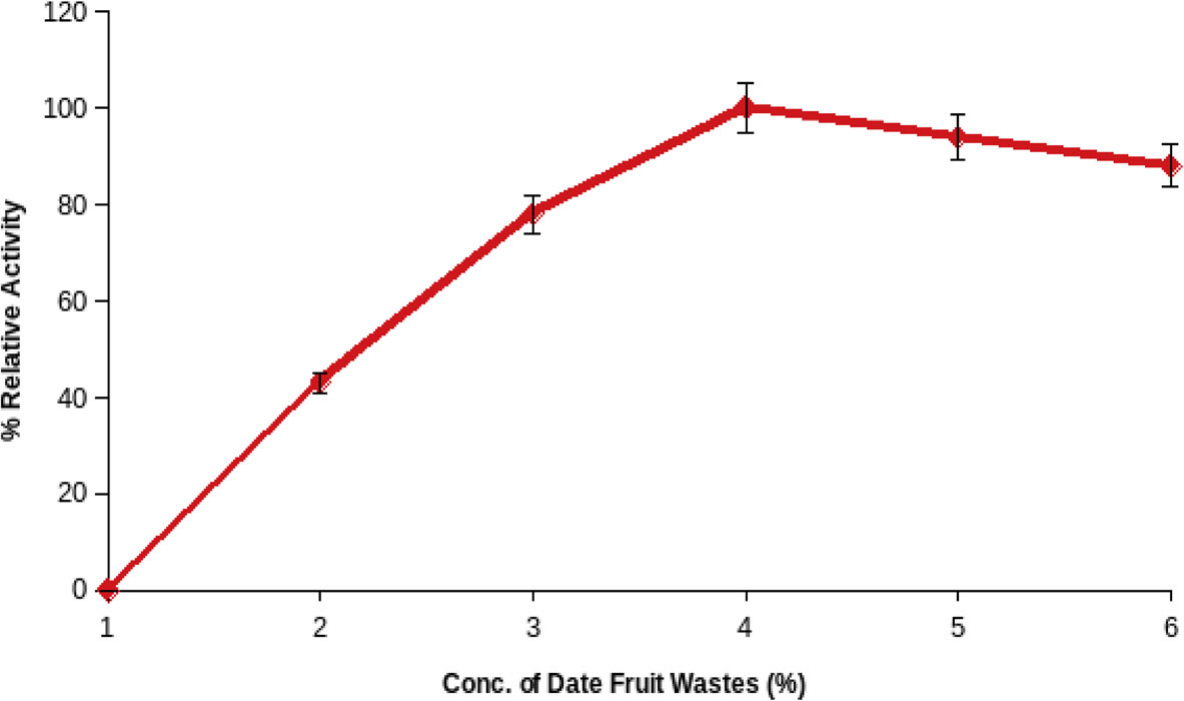
The influence of date fruit waste concentration on pectinase production by *B. licheniformis* KIBGE-IB3 (means ± S.E., *n* = 6)

The effect of date fruit waste concentration on the production of pectinase from *B. licheniformis* KIBGE-IB3 was analyzed using various concentrations of date fruit wastes ranging from 0 to 10% in fermentation medium. The production of pectinase from *B. licheniformis* KIBGE-IB3 was increased by increasing the date fruit waste concentration, and maximum production was achieved in the medium containing 5.0% of date fruit wastes (Fig. 1). Further increase of date fruits beyond 5.0 g/dl concentration decreased the pectinase production. No production of pectinase was seen at 0% of date fruit wastes.

#### Influence of nitrogen sources on pectinase production

**Fig. 2:**
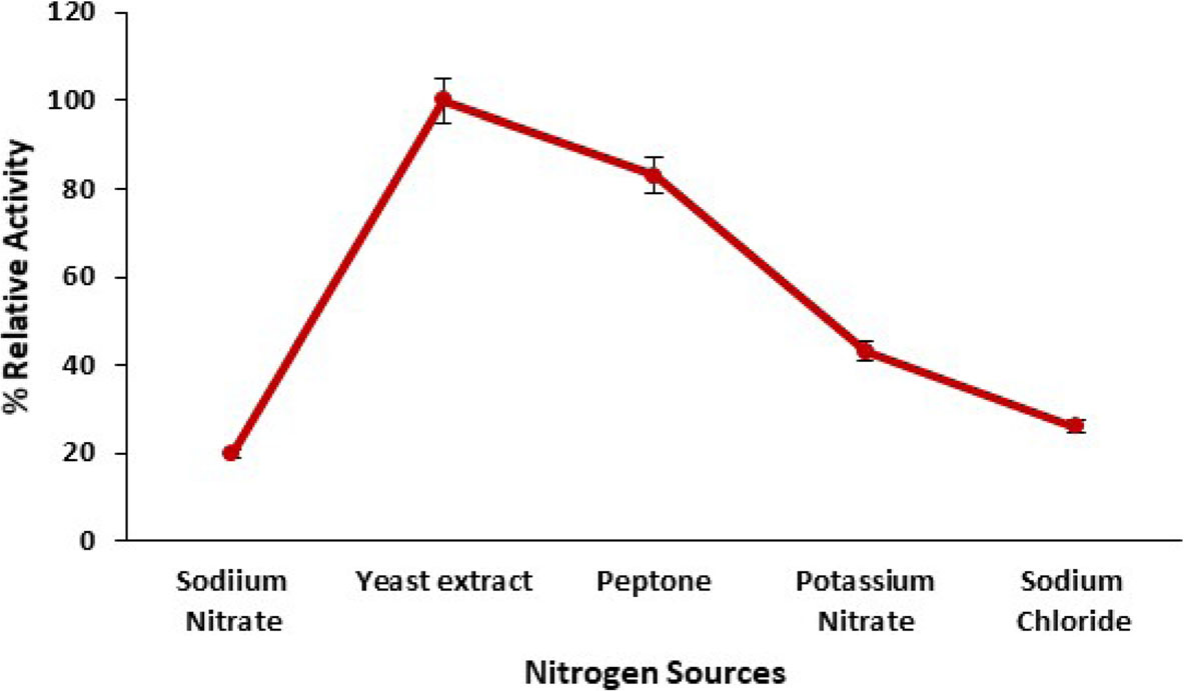
The influence of nitrogen sources on pectinase production by *B. licheniformis* KIBGE-IB3 using date fruit wastes as substrate (means ± S.E., *n* = 6)

The effect of nitrogen source on the production of pectinase from *Bacillus licheniformis* KIBGE-IB3 was analyzed by using a different type of nitrogen source in fermentation media, separately. It was observed that *Bacillus licheniformis* KIBGE-IB3 produced higher pectinase in yeast extract containing fermentation medium as compared to the other nitrogen sources (Fig. 2). The inorganic nitrogen sources reduced the pectinase production up to 70–50% with the comparison of yeast extract.

#### Influence of incubation time on pectinase production

**Fig. 3:**
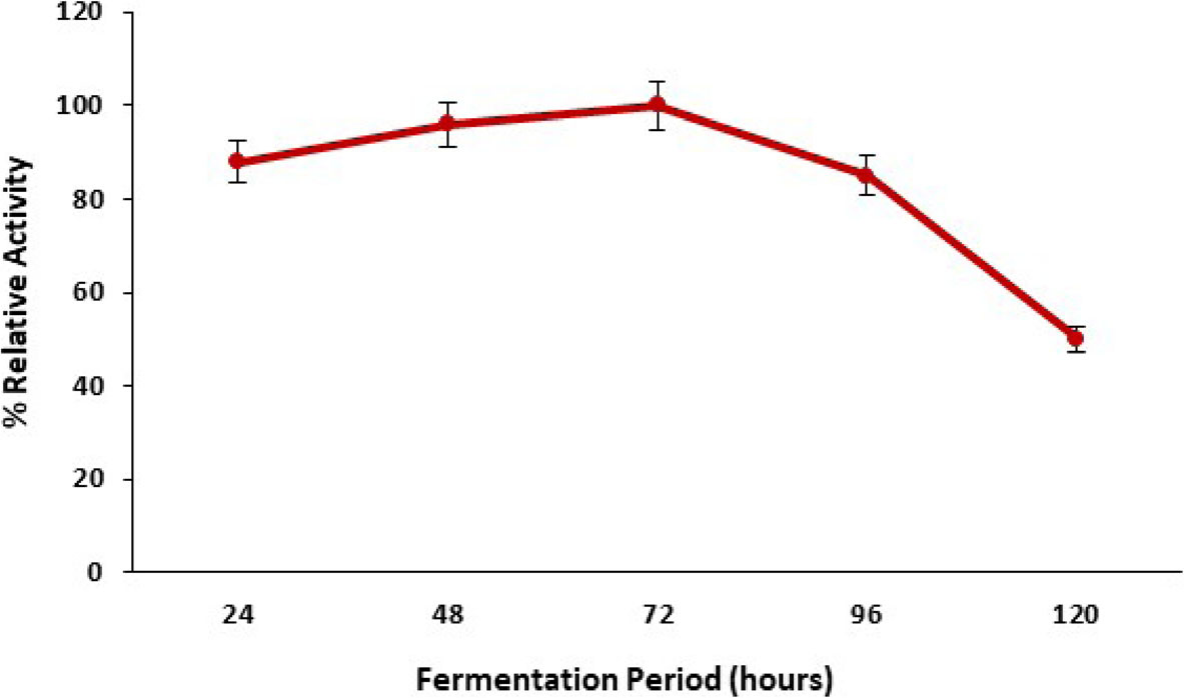
The influence of fermentation period on pectinase production by *B. licheniformis* KIBGE-IB3 using date fruit wastes as substrate (means ± S.E., *n* = 6)

The influence of incubation period on *Bacillus licheniformis* KIBGE-IB3 on the production of pectinase was monitored by fermenting the *Bacillus licheniformis* KIBGE-IB3 for different incubation times (24–120 h). *Bacillus licheniformis* KIBGE-IB3 started pectinase synthesis within 24 h, and the highest synthesis was obtained after 72 h of incubation (Fig. 3). The synthesis of pectinase was decreased when the fermentation period was increased to 120 h, and almost 15 to 50% pectinase synthesis was declined by *Bacillus licheniformis* KIBGE-IB3 at 96 and 120 h, respectively.

#### Influence of temperature on pectinase production

**Fig. 4:**
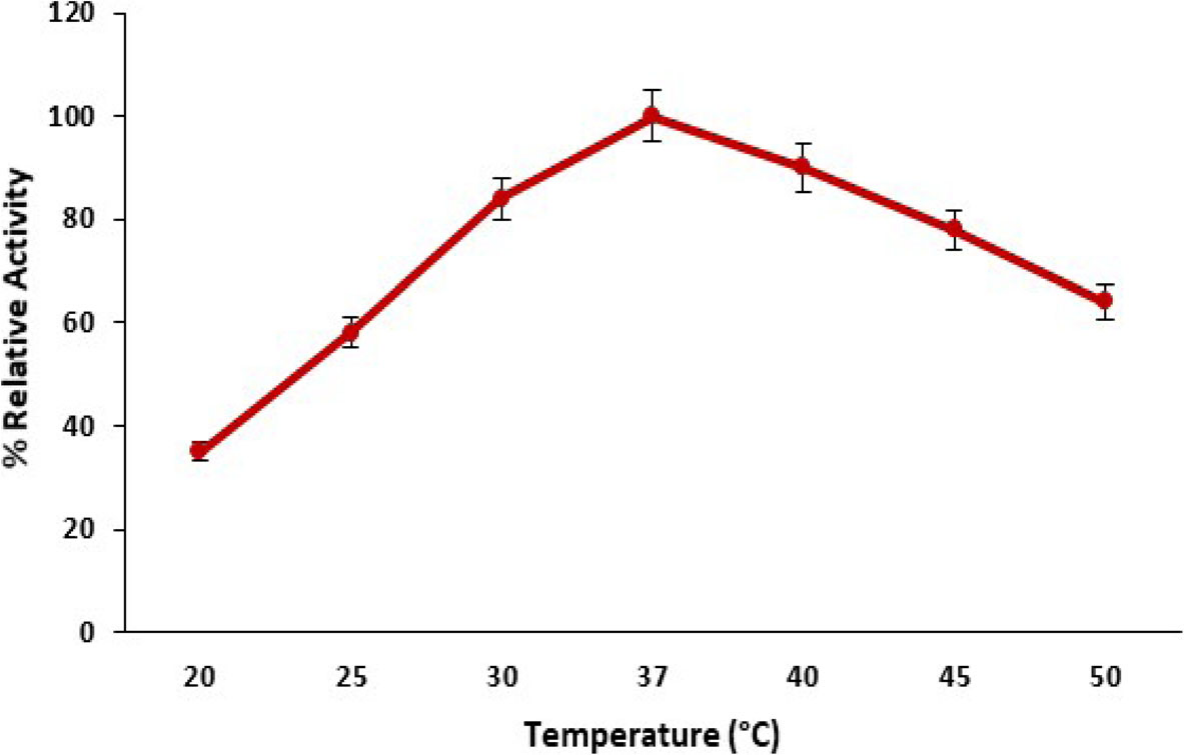
The influence of temperature on pectinase production by *B. licheniformis* KIBGE-IB3 using date fruit wastes as substrate (means ± S.E., *n* = 6)

The effect of incubation temperature on the production of pectinase from *B. licheniformis* KIBGE-IB3 was investigated by incubating the bacterial strains in different incubation temperatures. The biosynthesis of pectinase from *Bacillus licheniformis* KIBGE-IB3 was improved when the temperature was increased from 20 to 30 °C, and the highest biosynthesis of pectinase was obtained when the bacterial strain is incubated at 37 °C (Fig. 4). The pectinase biosynthesis was reduced to 36 to 10% when the temperature was increased to 40 and 50 °C, respectively.

#### Influence of pH on pectinase production

**Fig. 5:**
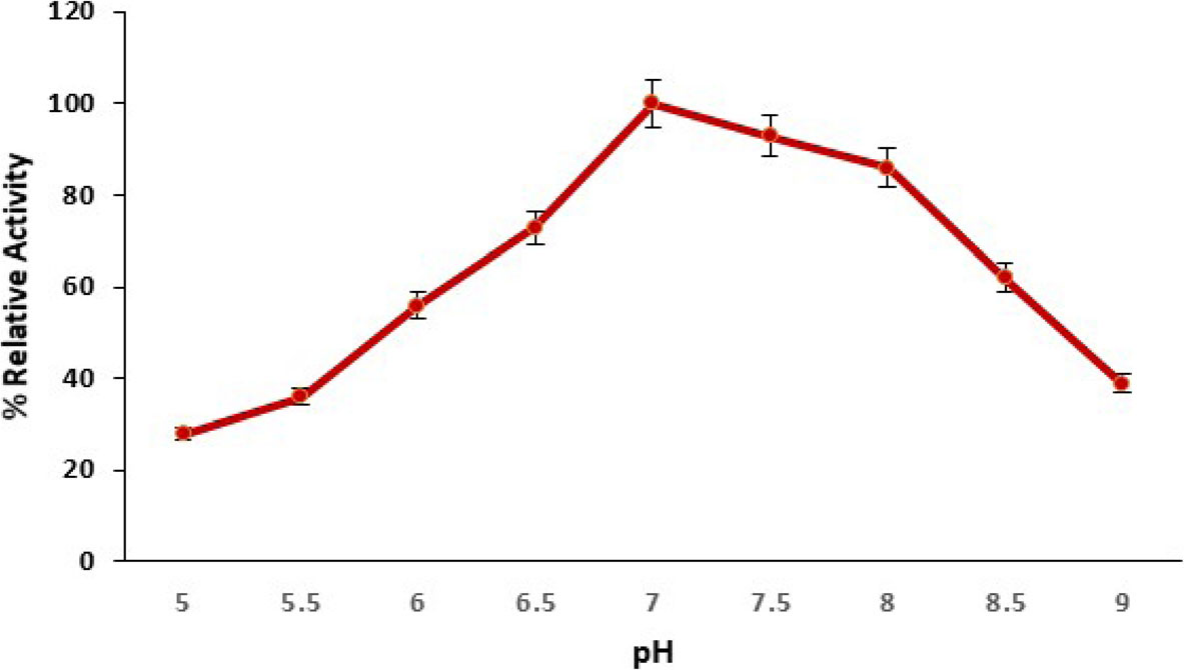
The influence of pH on pectinase production by *B. licheniformis* KIBGE-IB3 using date fruit wastes as substrate (means ± S.E., *n* = 6)

*Bacillus licheniformis* KIBGE-IB3 was inoculated in different media with different pH (5.0–9.0) at constant temperature and date fruit waste concentration for 72 h incubation. *Bacillus licheniformis* KIBGE-IB3 biosynthesized pectinase at a broad pH range (5–8) (Fig. 5). The pectinase biosynthesis was gradually increased by increasing the initial pH of fermentation medium and biosynthesized higher pectinase at pH 7.0. The variation of initial pH of fermentation medium beyond 7.0 either towards acidic (6.0 to 5.0) or alkaline (8.0 to 9.0) side reduced the pectinase biosynthesis.

## Discussions

The *Bacillus licheniformis* KIBGE-IB3 collected from KIBGE microbial bank was screened for pectinase, xylanase, amylase, and cellulase production using date fruit wastes as substrate by the SSF method. The *Bacillus licheniformis* KIBGE-IB3 produced higher pectinase as compared to cellulase, amylase, and xylanase using date fruit wastes. The higher production of pectinase by *B. licheniformis* KIBGE-IB3 using date fruit wastes may be due to the composition wastes, microbial sources, or genetic composition of bacterial strains. The utilization of date fruit wastes is for the production of value-added products by microbial species [11].

Fermentation technology is influenced by various growth factors such as composition of fermentation medium, temperature, pH, and incubation period. These physical and chemical factors are prime important for commercial production of enzymes, and various protocols have been suggested for enzyme production by microbial species [12, 13]. We used one variable at a time approach used for pectinase production by *B. licheniformis* KIBGE-IB3.

The *B. licheniformis* KIBGE-IB3 produced maximum pectinase using 5.0 g/dl date fruit wastes as substrate by solid state fermentation technology. Further increase of date fruit waste concentration reduced the pectinase production due to substrate-based inhibition and reduction of cell multiplication. The type of substrate and its concentration are very important for the manufacturing of microbial enzymes. It does not only work as a carbon source but also acts as inducer for specific enzyme production [14]. The production of pectinase was inducible in nature, and *B. licheniformis* KIBGE-IB3 did not produce pectinase without having substrate as date fruit wastes. The yeast extract with its complex composition supports the pectinase production, and *B. licheniformis* KIBGE-IB3 produced higher pectinase using yeast extract as a nitrogen source. The yeast extract contained various metal ions, proteins, vitamins, and other organic compounds which may fulfill the physiological needs of *B. licheniformis* KIBGE-IB3 for pectinase production. The yeast extract as a nitrogen source also induced the pectinase production from fungal species [15]. The microbial strains usually produced higher enzymes using complex organic nitrogen sources as compared to inorganic nitrogen sources [16]. The addition of yeast extract, peptone, and ammonium chloride in fermentation medium increased the pectinase production from *Bacillus* sp. DT7, while glycine, urea, and ammonium nitrate inhibited the pectinase production [17]. Peptone has been also found as a good organic nitrogen source for pectinase production form *Bacillus firmus*-I-10104 [12]. *Bacillus licheniformis* KIBGE-IB3 biosynthesized maximum pectinase after 72 h fermentation. *Bacillus sphacricus* (MTCC 7542) also produced pectinase. Similar findings were reported earlier regarding the synthesis of pectinase from [18]. The culture conditions and composition of media are important to maintain the microbial growth, and *Bacillus licheniformis* KIBGE-IB3 sustained a log phase within 24 to 72 h in terms of pectinase production. The reduction of pectinase production after increasing the fermentation time beyond 72 h of incubation might be due to exhaustion of substrate concentration in fermentation medium and the accumulation of toxic byproduct. It has been reported that the *Bacillus firmus*-I-10104 produced maximum pectinase after an incubation of 96 h [12].

Temperature is a very important factor for microbial enzyme production, and the biosynthesis of pectinase was improved by increasing the temperature from 20 to 37 °C. *B. licheniformis* KIBGE-IB3 biosynthesized higher pectinase at 37 °C. The biosynthesis of pectinase from bacterial strains was usually occurred within 37 °C [19]. *Bacillus licheniformis* KIBGE-IB3 synthesized pectinase at a higher temperature as compared to other microbial species which usually synthesized pectinase at 30 °C [18, 20]. The *Bacillus licheniformis* KIBGE-IB3 is mesophilic in behavior for pectinase biosynthesis.

The pH has a significant role for biosynthesis of enzymes [21]. The *Bacillus* species were usually synthesis enzymes from neutral to alkaline pH ranging from pH 7.0 to pH 9.0 [22–24]. *Bacillus licheniformis* KIBGE-IB3 biosynthesized pectinase at a broad pH range (5–8) and synthesized higher pectinase at pH 7.0. *Bacillus licheniformis* KIBGE-IB3 is neutrophilic in nature for pectinase production. The bacterial strains usually have optimum pH around neutral or alkaline for pectinase production, and fungi produce pectinase from neutral to acidic pH range. *Bacillus sp*. DT7 showed similar results and maintained maximum pectinolytic activity at neutral pH [25]. *Aspergillus niger* produced maximum pectinase at pH 6.5 [26], while *Aspergillus fumigatus* biosynthesized high pectinase at pH 4.0 [27].

## Conclusion

In the current study, *Bacillus licheniformis* KIBGE-IB3 was screened for the synthesis of amylase, xylanase, cellulase, and pectinase using date fruit wastes as substrate by solid state fermentation. *Bacillus licheniformis* KIBGE-IB3 produced higher pectinase using date fruit wastes as substrate as compared to other xylanase, cellulase, and amylase. The physical and chemical parameters of *B. licheniformis* of solid state fermentation were optimized for maximum pectinase production. *B. licheniformis* synthesized maximum pectinase using 5.0 g/dl date fruit wastes and 0.5 g/dl yeast extract. The production of pectinase reached maximum by keeping the fermentation of *B. licheniformis* KIBGE-IB3 at pH 7.0 and 37 °C for 72 h of incubation. *B. licheniformis* showed practicable properties for pectinase production using date fruit wastes. Further studies are needed to analyze the commercial feasibility of utilization of date fruit wastes for synthesis of enzymes as well as for the production of other value-added products.

## Data Availability

Available on request.
